# Relating excess spike synchrony to LFP-locked firing rates modulations

**DOI:** 10.1186/1471-2202-14-S1-P150

**Published:** 2013-07-08

**Authors:** Michael Denker, Alexa Riehle, Markus Diesmann, Sonja Grün

**Affiliations:** 1 Institute of Neuroscience and Medicine (INM-6) and Institute for Advanced Simulation (IAS-6), Jülich Research Centre and JARA, Jülich, Germany; 2 Institut de Neurosciences de la Timone (INT), CNRS-Aix-Marseille University, Marseille, France; 3 RIKEN Brain Science Institute, Wako City, Japan; 4 Theoretical Systems Neurobiology, RWTH Aachen University, Germany

## 

Synchronously firing groups of neurons, or cell assemblies, exhibit excess pair-wise synchronous spiking in parallel recordings of neuronal activity. Experimental work (e.g., [1]) indirectly substantiates the assembly idea with findings of behavioral correlates of significant spiking synchrony. We demonstrated [2] that such excess coincident spikes, or Unitary Events (UEs; [3]), show strong phase-locking to population oscillations exhibited by the local field potential (LFP), which cannot be explained by the spike-LFP relationship of individual neurons. A theoretical model links the observed coincidence-LFP relationship to the conceptual assembly framework. Co-varying firing rate modulations that correlate with LFP oscillations could represent an alternative cause for the observed phase-locking. In this scenario, the detected UEs occur as false positives due to firing rate non-stationarity (e.g., [4]). Here, we present two extensions of previous work [5] to check if this alternative hypothesis can as well explain our results.

In a first approach we propose an analysis to identify the largest rate changes in UE analysis windows under the assumption that they represent a rate step that is time-locked to a particular phase angle of the instantaneous LFP oscillation rhythm on a *trial-by-trial *basis. Using the difference of phases at which maximal rate changes are observed in two neurons we then relate the magnitude of individual rate steps to the degree of rate co-variation across neurons. By comparing the findings to theoretical calibrations tuned to the experimental rates, we find that although the firing rate modulation is related to LFP phase, the locking of significant spike synchrony does not preferentially occur with large co-varying changes of rate.

Based on these observations, we replace the parametric distribution of expected coincidences used in the original study [2] for evaluating the significance of observed coincidence counts by numerically derived distributions based on surrogate data that implement the null hypothesis that the LFP reflects the change in neuronal firing rate. To this end, we present a new surrogate method that dithers spike trains uniformly in operational time (see, e.g., [6]), a transformation of the original time axis given by the LFP time series. We are able to confirm our previous findings [2] on the relationship between precise spike synchrony and the LFP using this and other [4,5] surrogate methods, despite their decreased sensitivity in detecting excess spike synchrony. In summary, we demonstrate by these complementary analysis approaches that the original interpretation of LFP oscillations as a reflection of assembly dynamics is valid.
